# A Preliminary Study on the Whole-Plant Regulations of the Shrub *Campylotropis polyantha* in Response to Hostile Dryland Conditions

**DOI:** 10.3390/metabo14090495

**Published:** 2024-09-13

**Authors:** Hua Zhang, Xue Jiang, Lijun Zhu, Lei Liu, Zhengqiao Liao, Baoguo Du

**Affiliations:** 1 College of Urban and Rural Development and Planning, Mianyang Normal University, Xianren Road 30, Mianyang 621000, China; huazhang@mtc.edu.cn; 2 Engineering Research Center for Forest and Grassland Disaster Prevention and Reduction, Mianyang Normal University, Mianxing Road West 166, Mianyang 621000, China; jiangx079@mtc.edu.cn; 3 College of Life Science and Biotechnology, Mianyang Normal University, Mianxing Road West 166, Mianyang 621000, China; zhulijun211@mtc.edu.cn (L.Z.); liulei@mtc.edu.cn (L.L.); 4 Ecological Security and Protection Key Laboratory of Sichuan Province, Mianyang Normal University, Mianxing Road West 166, Mianyang 621000, China; 5 Chair of Ecosystem Physiology, Faculty of Environment and Natural Resources, University of Freiburg, Georges-Köhler-Allee 53, 79110 Freiburg, Germany

**Keywords:** drylands, metabolome, carbohydrates, drought, climate change, nitrogen, roots, partitioning

## Abstract

Drylands cover more than 40% of global land surface and will continue to expand by 10% at the end of this century. Understanding the resistance mechanisms of native species is of particular importance for vegetation restoration and management in drylands. In the present study, metabolome of a dominant shrub *Campylotropis polyantha* in a dry-hot valley were investigated. Compared to plants grown at the wetter site, *C. polyantha* tended to slow down carbon (C) assimilation to prevent water loss concurrent with low foliar reactive oxygen species and sugar concentrations at the drier and hotter site. Nitrogen (N) assimilation and turn over were stimulated under stressful conditions and higher leaf N content was kept at the expense of root N pools. At the drier site, roots contained more water but less N compounds derived from the citric acid cycle. The site had little effect on metabolites partitioning between leaves and roots. Generally, roots contained more C but less N. Aromatic compounds were differently impacted by site conditions. The present study, for the first time, uncovers the apparent metabolic adaptations of *C. polyantha* to hostile dryland conditions. However, due to the limited number of samples, we are cautious about drawing general conclusions regarding the resistance mechanisms. Further studies with a broader spatial range and larger time scale are therefore recommended to provide more robust information for vegetation restoration and management in dryland areas under a changing climate.

## 1. Introduction

In the context of global climate change, extreme drought and heat events are projected to happen more frequently, which have brought particularly negative impacts to ecological vulnerable regions [1,2,3]. The dry-hot valleys (DHVs), located along the upper streams of many rivers and characterized by both arid conditions and high temperatures, are at the frontline of these climate change impacts [4,5]. The plants inhabiting DHVs are thus supposed to experience more severe drought and heat episodes exacerbated by climate change. However, so far, the studies on plants from DHVs are mainly focused on general adaptation traits such as growth measurements, leaf morphological and photosynthetic traits [6,7,8], very few studies have yet shed lights on how such specific DHV climate affects the carbon and nitrogen metabolism, especially how plants coordinate the metabolic processes between the root and leaf tissues during the long-term adaptation to the local environment.

Drought and heat stresses, whether occurring individually or together, are among the most common stresses confronted by plants in nature, especially in DHVs [9]. During the growth season, both drought and heat stresses can result in significant changes in physiological processes involved in carbon and nitrogen metabolism and eventually affect the growth and development of the stressed plants [10,11,12]. For example, extended drought treatment tended to restrict root nitrate update and further reduce the nitrate transportation from root to leaf tissues [13]; while heat stress further decreased the root update of nitrogen, phosphate and potassium and eventually caused less availability of these macronutrients to the leaf tissues, hence decreased growth of the shoot [14]. The shoots, in particular the leaves on the other hand, are forced to adjust their stomata as a response to the severe dehydration and high leaf temperature caused by drought and heat stresses, which often lead to accumulation of reactive oxygen species (ROS) in chloroplast and further damages on the photosynthetic apparatus [15]. To cope with the stressful conditions, plants often accumulate compatible solutes (e.g., sugars and amino acids) and antioxidants to maintain turgor pressure and keep ROS levels under control [15,16,17,18].

At the whole-plant level, both leaves and roots are critical to plant survival and fitness; however, they differ in their resource uptake and metabolic roles and, often, in their vulnerability and response to stresses [15,16]. Particularly in drylands, despite harsh drought and the high temperature environment, the longer growth season for the vegetation may extend the period for biomass accumulation in both leaf and root tissues, thus both the primary and secondary metabolism are enhanced in these plants. Since the leaf senescence is a long-term developmental and important nutrient management process by the end of the growth season, the mobilization of cell constituents including amino acids, sugars and lipids derived from the chloroplast degradation to the sink tissues prepare the plants well for the next growth season [19,20]. Such a process, however, might be quickly triggered and even brought forward by extended drought and high temperature events in such specific DHV climates. Yet, so far, no studies have ever underlined the patterns of transportation and distribution of metabolites between the roots and leaves of the dominant plant species of DHVs before leaf fall and the regulation mechanisms under the DHV climate.

*Campylotropis polyantha* is one of the pioneer shrub species widely distributed in the arid Minjiang River valley in Southwestern China, one of the most important and fragile ecological zone along the Yangtze River [6,8]. They play very important ecological roles to protect the dryland from degradation. Previous studies have shown well-adapted morphological and anatomical traits of *C. polyantha* leaves to arid conditions and demonstrated its high potentials to cope with future severe drought and heat conditions [6,8]. However, little is known about the metabolic regulations of this dominant shrub species in response to arid DHV conditions. In the present study, we investigated the water relations, reactive oxygen species levels and metabolome of *C. polyantha* plants from two sites with distinct soil water and temperature conditions along the Minjiang River DHV. Specifically, we hypothesized that, (1) compared to the plants inhabiting in the wet and cool site, plants in the drier and hotter site had lower tissue relative water contents but higher ROS levels; (2) metabolites with antioxidant and osmoprotective properties accumulated in plants at the drier and hotter site, which was concurrent with declined C content due to limited C assimilation; (3) and different metabolite distribution patterns between leaves and roots in response to site conditions; specifically, the plants at the drier and hotter site may advance the metabolites transportation from leaves to roots before senescence.

## 2. Materials and Methods

### 2.1. Field Sites

The study area is located in the dry and hot valley (31°26′–33°16′ N, 102°59′–104°14′ E) of the upper Minjiang River (Minjiang DHV), Sichuan province, Southwest China, with typical semi-arid continental monsoon climate as described in Liao et al. [21]. The mean annual precipitation, evaporation and temperature are 495 mm, 1332 mm and 11.2 °C, respectively. The average sunshine duration was 1675 h. Within this region, two field sites with distinct climate conditions but similar vegetations were selected, i.e., Lianghekou (1820 m above sea level) and Cuojishan (1655 m above sea level) representing arid (AR) and optimal (OPT) conditions, respectively. The arid site of Lianghekou is located in the core area of the Minjiang DHV with strong foehn effect, where the mean annual precipitation and air temperature were 369 mm and 13.3 °C, respectively. The annual evaporation at Lianghekou reached 1883 mm. The optimal site of Cuojishan is located in the edge of the Minjiang DHV, where the mean annual precipitation and air temperature were 574 mm and 10.6 °C, respectively [22]. No extreme drought and heat events occurred in this area in the period 2019–2021 [23]. At the time of sampling (October 2021), the upper layer soil (0–20 cm) water content at the optimal site of Cuojishan was found to be 68% higher (*p* < 0.01) than that at the arid site of Lianghekou. More soil characteristics at the two sampling sites are described in Liao et al. [21].

### 2.2. Leaves and Roots Sampling

Within each site, leaf and root samples from six vigorous individual plants were harvested during the late growing season in October 2021. We chose this time for sampling to facilitate insight into the absorption and allocation of resources prior to the onset of leaf senescence. Leaf sample per plant was collected from at least three twigs. Roots of each plant were carefully dug out and fine roots (diameter < 2 mm) were harvested after clean with distilled water and dried with cellulose paper. Plant samples were immediately frozen in liquid nitrogen. The plant samples were transferred to lab under frozen and homogenized with mortar and pestle in liquid nitrogen and then stored at −80 °C until biochemical analysis. Leaf and root relative water contents were determined using the frozen materials after oven drying. The relative water content of plant tissue was calculated with the following equation.

relative water content (g H_2_O g^−1^ DW) = (FW − DW)/DW,

where FW and DW represent the fresh weight and dry weight of plant material.

### 2.3. Biochemical Analyses

#### Biochemical Analyses of Plant Samples

Plant hydrogen peroxide (H_2_O_2_) was extracted in 0.1% (*w*/*v*) trichloroacetic acid and the absorbance was determined at 390 nm after reaction with 1 mol KI as previously described in Du et al. [24]. Total carbon (C), total nitrogen (N) contents, C and N isotopes signatures were measured with elemental analyzer–isotope ratio mass spectrometer (EA-IRMS) (EA1112 coupled with Delta-XP, Thermo Fisher Scientific, Bremen, Germany) as previously described [25]. Glutamic acid was analyzed as working standard for calibration. The ^13^C and ^15^N abundances of samples are expressed relative to the international reference VPDB and atmospheric N_2_, respectively.

^13^C_sample_ = [((^13^C/^12^C)_sample_)/((^13^C/^12^C)_VPDB_) − 1] × 1000

^15^N_sample_ = [((^15^N/^14^N)_sample_)/((^15^N/^14^N)_atmospheric N2_) − 1] × 1000

Plant soluble protein was extracted in Tris-HCl buffer, and total soluble protein contents were quantified using the colorimetric Bradford method as previously described [26]. Bovine serum albumin was used as standard for quantification. Amino acids of sample were extracted with 1 mL of chloroform/methanol mixture (1.5/3.5, *v*/*v*) as previously described [26]. Total amino acid content of the extract was colorimetrically determined at 570 nm after derivatization with ninhydrin reagent as described by Du and Rennenberg [27]. Total soluble sugar contents were calorimetrically determined using the anthrone–sulfuric acid method [27].

Water-soluble low-molecular-weight polar metabolites were extracted from approximately 50 mg frozen powder in 600 µL pure methanol with heating at 70 °C for 10 min. Supernatant of 500 µL was combined with the same volume of *dd*H_2_O and cold chloroform. After centrifugation, an aliquot of 100 μL of the supernatant from the methanol phase was freeze-dried for 48 h. For derivatization, the dried extract was dissolved in 20 μL of a 20 mg mL^−1^ solution of methoxyamine hydrochloride in anhydrous pyridine, and the solution was incubated at 30 °C for 90 min with constant shaking. Thereafter, 40 μL N-methyl-N-(trimethylsilyl)-trifluoroacetamide (MSTFA, Sigma-Aldrich Chemie, Shanghai, China) was added and the mixture was incubated at 37 °C for 30 min with shaking. Then, 50 μL solution was transferred into glass vials with inserts and sealed for GC-MS analysis. Samples were measured using a Thermo gas chromatograph coupled with a Thermo mass spectrometer detector (ISQ™ 7610 Single Quadrupole GC-MS system, Thermo Fisher Scientific). The GC-MS analysis was performed using the parameters previously described [24]. Aliquots of 1 μL derivatized sample were injected in splitless mode into the system and separated on a TG-5 MS capillary column (30 m × 0.25 mm ID, 0.25 µm film thickness, Thermo Fisher Scientific). The mass spectrometer was operated in electron ionization mode (EI) at 70 eV with a scan range of 50 to 500 m/z. Peak detection and alignment were performed with the free AMDIS (automated mass spectral deconvolution and identification system, version 2.7) software supplied by NIST (National Institute of Standards and Technology, Gaithersburg, MD, USA). Metabolites were normalized using the peak area of the internal standard, ribitol, and the dry weight of samples and presented as relative abundances. Signals corresponding to artifacts were omitted according to the analysis of ‘blank’ samples prepared in the same manner as biological samples. Unknown compounds were excluded from further analyses.

### 2.4. Statistical Analysis

Statistical analyses were performed using SPSS 22.0 (International Business Machines China Co., Ltd., Chengdu, China). Differences between the two sites within the same tissue were determined by Student’s *t*-test. Data were transformed by denary logarithm to match normal distribution when necessary. Data shown in figures represent means ± SD of 6 plants (*n* = 6) on a dry weight basis. Principal component analysis (PCA) was conducted using a public web tool (MetaboAnalyst 6.0, https://www.metaboanalyst.ca/MetaboAnalyst/, accessed on 15 June 2024) [28] after log10 transformation and mean centering. Missing values were replaced by 1/5 of minimum abundance of respective compounds, assuming that their concentrations were below detection limit. Venn diagram was created on an open webtool (https://bioinformatics.psb.ugent.be/webtools/Venn/, accessed on 30 June 2024).

## 3. Results

### 3.1. Differences in Leaves between the Two Sites

**Figure 1:**
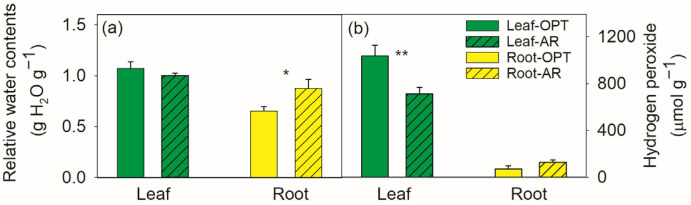
Contents of water (**a**) and hydrogen peroxide (**b**) in leaf (green bars) and root (yellow bar) of *Campylotropis polyantha* at site with optimal conditions (OPT, without hatching) and site with arid conditions (AR, hatched bars). Asterisks indicate significant differences between the two sites within the same tissue (*, *p* < 0.05; **, *p* < 0.01). Data are presented as means ± SE (*n* = 6) on a dry weight basis.

Compared to plants in optimal conditions (OPT), plants in arid conditions (AR) had similar foliar water contents (Figure 1a) but 31% lower hydrogen peroxide (H_2_O_2_) contents (Figure 1b).

**Figure 2:**
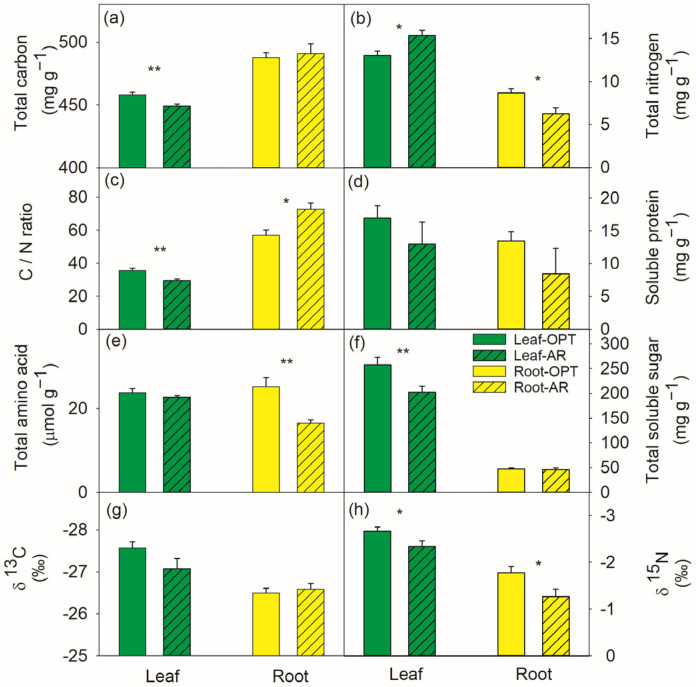
Total carbon (**a**), nitrogen (**b**) contents and their ratios (**c**), soluble protein (**d**), total amino acid (**e**), soluble sugar (**f**), δ^13^C (**g**) and δ^15^N (**h**) in leaf (green bars) and root (yellow bar) of *Campylotropis polyantha* at site with optimal conditions (OPT, without hatching) and site with arid conditions (AR, hatched bars). Asterisks indicate significant differences between the two sites within the same tissue (*, *p* < 0.05; **, *p* < 0.01). Data are presented means ± SE (*n* = 6) on a dry weight basis.

The leaves of plants in AR had a 2% lower total C (*p* < 0.01) but an 18% higher total N contents (*p* = 0.029), and a declined C/N ratio (*p* < 0.01), compared to OPT (Figure 2a–c). No significant site-specific differences in the leaf soluble protein and the total amino acid concentrations were observed (Figure 2d,e). The total soluble sugar contents were 21% lower in the leaves of plants in AR (Figure 2f). Leaf δ^13^C (*p* = 0.06) and δ^15^N (*p* = 0.03) were less enriched in AR (Figure 2g,h).

**Figure 3:**
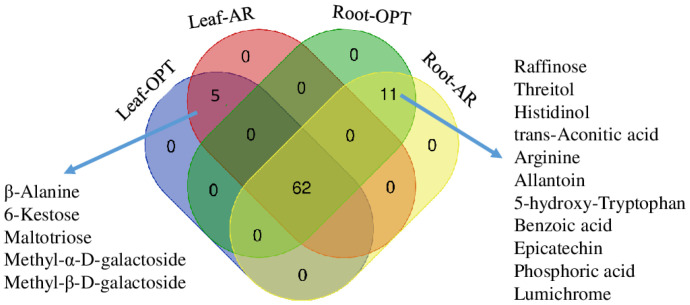
Venn diagram showing metabolites abundant in leaves and roots of *Campylotropis polyantha* at site with optimal conditions (OPT) and site with arid conditions (AR). The numbers indicate the number of shared compound(s) of the groups.

**Figure 4:**
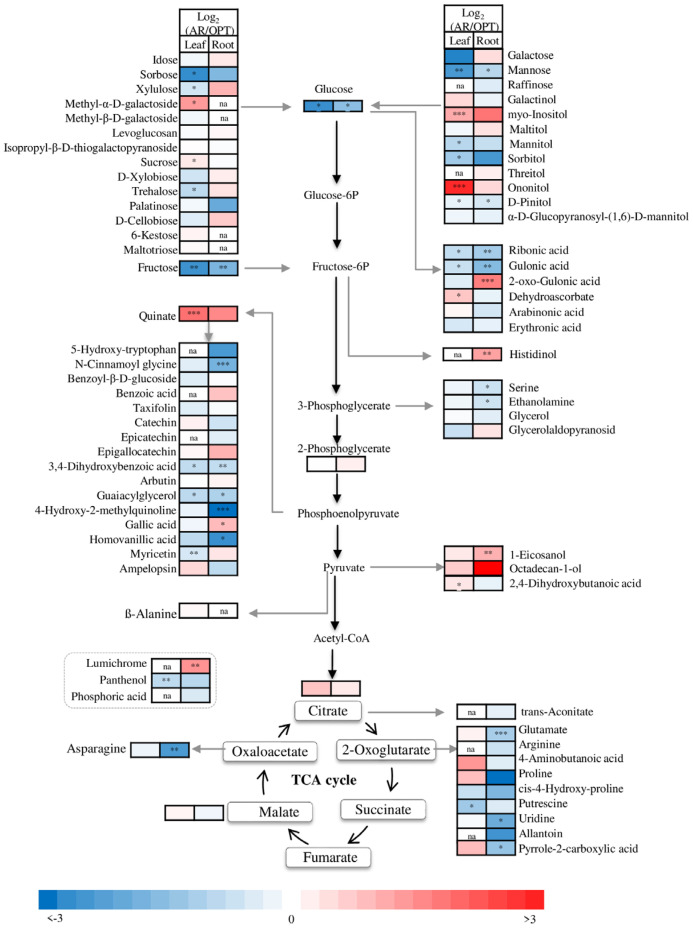
Fold change (log_2_) of metabolites in leaves (**left panels**) and roots (**right panels**) of *Campylotropis polyantha* between the arid site (AR) and the optimal site (OPT), respectively. Asterisks indicate significant differences between sites within the same tissue, and between tissues within the same site (*, *p* < 0.05; **, *p* < 0.01; ***, *p* < 0.001; na, the metabolite was not detectable). Data are presented means ± SE (*n* = 6) on a dry weight basis.

In total, sixty-seven metabolites were identified in leaves including seventeen sugars (ten monosaccharide, five disaccharides, two trisaccharides), three sugar derivates, nine organic acids, seven amino acids, five nitrogen compounds, fourteen aromatics, eighth polyols, one phosphorus compound, two fatty alcohols and one fatty acid (Figure 3 and Table S1). There were clear differences in these metabolites in leaves between the two sites. Foliar sugars concentrations of plants in AR were generally lower than plants in OPT, particularly the monosaccharides of glucose, fructose and mannose (Figure 4). In contrast to sucrose with higher concentrations in plant leaves in AR, sorbose, xylulose and trehalose were significantly more abundant in leaves in OPT. Myo-inositol and ononitol were dramatically accumulated in leaves of plants in AR (*p* < 0.001), and similar effects were observed on dehydroascorbate (DHA). D-pinitol, mannitol and sorbitol were accumulated in leaves of plants in OPT (Figure 4). Foliar concentrations of ribonic acid and gluconic acid were significantly lower in plants in AR. In contrast to extremely (*p* < 0.001) accumulated quinate in leaves of plants in AR, aromatic compounds were less abundant, particularly for 3,4-dihydroxybenzoic acid, guaiacylglycerol and myricetin (Figure 4). No significant differences in foliar citrate, malate and amino acid concentrations between the two sites were observed.

### 3.2. Differences in Roots between the Two Sites

Compared to plants in OPT, the roots of plants in AR had a 33% higher water contents (*p* = 0.03) (Figure 1a) and similar H_2_O_2_ contents (Figure 1b). No significant site effects were observed on the root concentrations of the total C, soluble protein and total sugar, as well as δ^13^C. The roots of plants in AR had a 29% lower total N and 34% lower total amino acid contents. The root C/N ratio and δ^15^N were significantly increased in AR (Figure 2).

**Figure 5:**
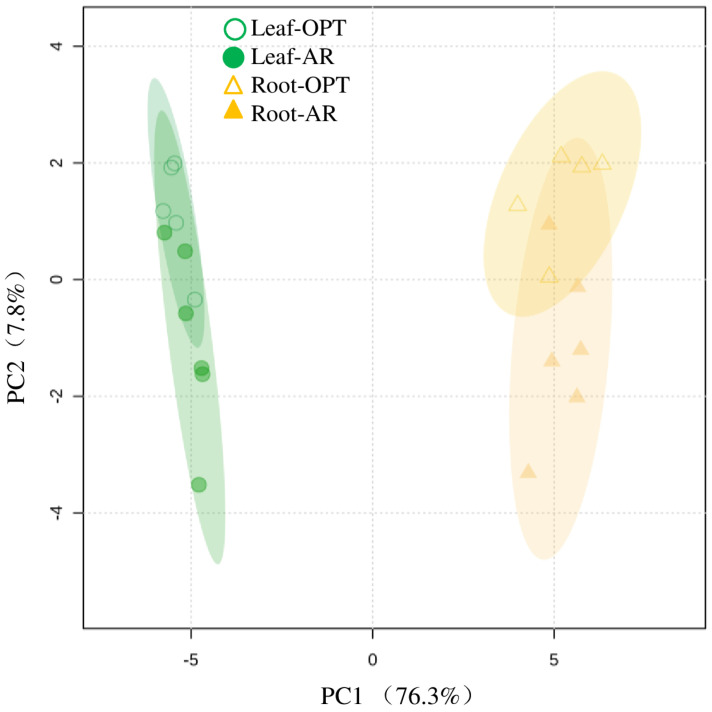
Clustering of all physiological and metabolic parameters in leaves (L, circle) and roots (R, triangle) of *Campylotropis polyantha* in the optimal site (OPT) and the arid site (AR). Semi-transparent shadings indicate 95% confidence regions.

In total, 73 metabolites were identified in roots, and 11 of them were not abundant in leaves (Figure 3 and Table S1). Like in leaves, the concentrations of glucose, fructose, mannose and D-pinitol were less abundant in the roots of plants in AR (Figure 4). No significant site effects on other sugars and polyols were observed. The root concentrations of ribonic acid and gluconic acid were significantly lower (*p* < 0.01) in plants in AR, whereas 2-oxo-gulonic acid was more abundant (*p* < 0.001) in the roots of AR plants. Except for histidinol and lumichrome, the concentrations of amino acids and other N compounds were largely declined in the root of plants in AR compared to plants in OPT (Figure 4), Similar site effects were observed in secondary metabolites except for galic acid. The two fatty alcohols were accumulated in roots in AR (Figure 4). PCA, including metabolome and all the other physiological parameters, showed clear clusters in response to different plant organs along component 1 and site effects on leaves and roots along component 2. The two components explained 84.1% of the total variance (Figure 5).

## 4. Discussion

Generally, shrubs are drought tolerant due to their extensive and deep root structures; therefore, they are often pioneer woody species in arid and semi-arid areas and play an important role in maintenance of dryland vegetation [29,30]. In the present study, the physiological responses of a dominant woody shrub species *Campylotropis polyantha* acclimated to a drier and hotter site (AR) were compared with plants grown at a wetter and cooler site (OPT) at the Minjiang dry and hot valley [6,8]. A sophisticated resistance mechanism involving ROS control, C and N metabolism at the whole-plant level has been revealed in *C. polyantha* plants under stressful conditions.

### 4.1. Water Relations, ROS Control and C, N Metabolic Regulations in Leaves

In line with previous studies showing high drought resistant [31], we found *C. polyantha* held constant leaf water content but lower hydrogen peroxide levels under dry and hot conditions (Figure 1). The progressive water stress-induced decreases in leaf hydration in *C. polyantha* seedlings [8] were not observed. Although more studies have shown H_2_O_2_ acts as a signaling molecule involved in many aspects of plant metabolism and stress response, high levels of H_2_O_2_ can cause oxidative damage and triggers programmed cell death. Therefore, its production and scavenge are well controlled by the plant [32,33]. The scavenging system includes enzymatic and non-enzymatic manners. In the present study, the dramatically accumulated polyols of myo-inositol [34,35] and its derivative of ononitol [36] may play important roles in controlling the ROS levels. Previous studies also shown that polyols as antioxidants and osmoprotectants play a crucial role in plants against a variety of abiotic stresses [37]. The ascorbate–glutathione cycle has a significant role in protecting plant cells against damage from high ROS levels [38], for instance, in woody shrub and conifer species [27,39,40]. In the present study, the stimulated ascorbate-glutathione cycle is projected as indicated by the significantly increased production of the oxidized form DHA in leaves at the hot and drier site (Figure 4), which is normally present at only trace concentration under optimal conditions [38]. Aromatic compounds are promising ROS scavengers, not only because of their unique benzene-based structures, but also because they enhance the antioxidant defense system [41,42]. Accumulated aromatic compounds were often observed [24,39,43,44] to provide tolerance in plants against various stresses [33,41,42]. In the present study, significantly increased aromatic compounds were not observed, although quinate was accumulated in AR compared to OPT site (Figure 4), which was in line with the low ROS levels and therefore more carbon can be directed to other metabolic process supporting growth without extra cost for remobilization and reallocation to growth and development upon demand [45].

Carbon (C) and nitrogen (N) are the two most abundance essential elements in plants and play critical roles in metabolism, growth and stress response. Sugars are essential to the fundamental processes required for plant growth and resistance. The disaccharides of sucrose, trehalose, raffinose family oligosaccharides and fructans are three major types of water-soluble carbohydrates essentially involved in plant stress responses [46]. They act as building units, energy providers, signal molecules, and the stored sugars provide an important source of metabolic substrate for plants during stress [47,48]. In the present study, total soluble sugar contents were 21% lower in leaves of plants at the hotter and drier site AR than plants in OPT; this was mostly due to the declined concentrations of fructose, glucose, sorbose, xylulose, trehalose, mannose, galactose and raffinose, which was under the detect limitation in leaves (Figure 3). The low sugar concentrations can be explained by the low photosynthesis of plants in AR, as seen from the significantly decreased total C and slightly enriched δ^13^C signature (*p* = 0.06) (Figure 1), due to decreased C isotope discrimination and therefore increased stomatal closure and higher water use efficiency [49,50]. This is in line with the decreased light harvesting complex and reduced photochemical processes and PSII activity under drought conditions and the lower number of stomata and epidermal cells per leaf surface unit at higher elevation [6,8]. Only methyl-α-D-galactoside and sucrose were accumulated in the leaves of plants in AR (Figure 4); the latter controls various developmental and metabolic processes in plants [51]. Drought-induced sucrose accumulation has also been documented in other plant species [18,52], probably due to the limited transport from leaves to roots, thus helping maintain leaf functions [51].

Plant C and N metabolisms are tightly correlated [53]. N and phosphorus (P) are often the limiting nutrients that constrain the magnitude of terrestrial carbon uptake, particularly in areas, such as dry lands, with low soil water availability and nutrients [54,55,56]. Therefore, a better understanding of N in plants is particularly important for elucidating physiological processes. Xiong et al. [56] recently found the dominant plants in China’s drylands generally displayed higher leaf C:N and C:P ratios than the global dataset [57], indicating that drought imposes greater constraints on N and P than on C in drylands [58]. Our study is in line with the large-scale patterns of plant C and N stoichiometry along environmental gradients, that aridity generally exerted negative effects on C and positive effects on plant N [56]. Concurrent with the declined foliar total C contents, sugars were less abundant in the leaves of plants in AR. Similar effects of drought on leaf C contents were observed in *C. polyantha* seedlings [7]. However, a drought-induced decrease in the leaf total N contents [7] were observed in the present study, probably indicating different responses of seedlings and mature plants [59]. In the present study, the foliar N content was significantly increased at the expense of the root N content in AR in comparison to OPT (Figure 2). The concentrations of leaf N-containing compounds, i.e., soluble protein, specific amino acids and total amino acid did not decline (Figure 2 and Figure 4). Therefore, the concentrations of other N-containing compounds, such as pigments [8], alkaloids and the most abundant protein on earth, Rubisco (d-ribulose 1,5-bisphosphate carboxylase/oxygenase) [60], in plants at different sites deserve further investigation. Plant δ^15^N values can reflect the δ^15^N characteristics in the soil N source [61] and the integrated process of N fluxes, assimilation and allocation. Greater δ^15^N generally indicates an increased inorganic N turnover and a higher efflux/influx ratio [62]. In the present study, the enriched δ^15^N in plants in AR apparently indicated that N uptake and assimilation were not impaired [62,63]. However, further studies are required to elucidate the N uptake and cycling of *C. polyantha* under arid environments. Positive correlation between foliar δ^15^N and aridity was also found across a wide spectrum of ecosystems [64]. Therefore, the present study also highlights the potential of *C. polyantha* as a nitrogen-fixing shrub species in dryland ecological restoration [6,8,65].

### 4.2. Water Relations, ROS Control and C, N Metabolic Regulations in Roots

As the first targets receiving soil changes, roots play a pivotal role in defining plant growth, development, ecological success and mediating terrestrial ecosystem functioning [66]. Both the phenotypic and biological changes in roots are of particular importance for plants to cope with hostile conditions [67]. Compared to the leaf or shoot, less attention has been paid on root physiology in response to changing climate conditions [66]. Understanding the contrasting metabolic changes in different parts of the plant in response to various climate conditions can help decipher strategies for coping with stressful conditions. The partitioning patterns can be different in response to growth stage and season.

In the present study, we found that *C. polyantha* at the hotter and direr site AR held constant hydrogen peroxide levels and had a 33% higher water content (*p* = 0.03) (Figure 1). Considering the similar hydration and significantly decreased H_2_O_2_ content in leaves in AR, our first hypothesis has to be rejected. Similarly, hydrated root tissues at the lower substrate water potential were documented in maize (*Zea mays* L.) seedlings [68]. However, no apparent accumulation of osmolytes or compatible solutes was found in the present study. Apart from the reduced transport from the roots to the shoot prepared for leaf senescence, the root-abundant and accumulated metabolites, i.e., raffinose, trehalose, D-xylobiose, threitol, histidinol and most N compounds derived from TCA cycle (Figure 3), may be at least partly involved to keep root hydration.

Compared to leaves with a higher total N and similar total amino acid contents in AR, the total N and total amino acid contents in roots were both lower in AR than OPT (Figure 2). The declined total amino acid content was mainly due to lower concentrations of serine, asparagine and amino acids from the glutamate family (Figure 3), indicating changes from assimilation to remobilization before the onset of senescence [69]. The different metabolomes between leaves and roots [70] can be explained by the already slowed-down metabolism of the whole plant and also the translocation to other storage pools like stems and coarse roots [7,8,31]. Therefore, our second hypothesis was only partly supported because no apparent accumulation of metabolites with osmoprotective properties in plants at the drier and hotter site AR was observed.

In the present study, climate conditions had only minor effects on the partitioning of metabolites between leaves and roots (Figures S1–S3). Therefore, our third hypothesis assuming the plants in AR may advance the metabolites transportation from leaves to roots before senescence has to be rejected. On the other hand, our results suggested that plants were still undergoing vigorous growth at both sites. The leaves of the plants in AR had generally higher contents of carbohydrates, sugar derivatives, except the three disaccharides and threitol. Amino acids except asparagine and other N compounds derived from the citric acid (TCA) cycle were less abundant in leaves than roots, whereas serine and β-alanine were significantly higher in leaves than roots (Figure 4). The different partitioning of N compounds may be due to the predominant allocation of limited C skeletons to the upper stream metabolic processes like sugar and secondary metabolisms, rather than to the precursor of citrate and the intermediates of the TCA cycle due to the reduced energy demand of the plants before the onset of senescence [71]. Similar TCA cycle-involved leaf senescence was also reported in other plant species [72].

## 5. Conclusions

In conclusion, understanding the whole-plant regulation in response to stressful environmental conditions can provide valuable information for vegetation restoration and management. This is of particular importance in dryland areas, where more droughts of unprecedented severity are projected in the context of climate change. Here, we investigated the leaf and root metabolome and water relations and ROS levels of a dominant woody shrub *C. polyantha* in a typical dry and hot valley in Minjiang, China before the onset of leaf senescence. As an adaptation to difficult environmental conditions, *C. polyantha* showed a well-tuned resistance strategy, including mechanisms to prevent water loss and ROS accumulation, and whole-plant metabolic regulations. To be aware, we harvested the samples only once in the growing season. In addition to water and temperature, other factors like the season, different solar radiation and growth periods between the two field sites may have also contributed to the observed metabolomic differences. Due to the small sample size, we cannot draw strong conclusions. Thus, we recommend studies with more intensive sampling points and experiments under controlled conditions to disentangle the effects of specific factors. The differently allocated nitrogen and secondary compounds highlight our incomplete understanding of plants’ acclimation to climate change. Therefore, further studies are necessary to elucidate the adaptations of nitrogen uptake and assimilation, the tradeoff between growth and defense, and the assimilation and allocation between the primary and secondary metabolisms of plants under stressful conditions.

## Data Availability

The data presented in this study are available on request from the corresponding author.

## Supplementary Materials

The following supporting information can be downloaded at: https://www.mdpi.com/article/10.3390/metabo14090495/s1, Table S1: List of identified compounds in leaves and roots of *Campylotropis polyantha*. Figure S1: Water and hydrogen peroxide contents in leaf and root of *Campylotropis polyantha* at the two sites. Figure S2: Total carbon, nitrogen contents and their ratios, soluble protein, total amino acid, and soluble sugar contents, and δ^13^C and δ^15^N signatures in leaf and root of *Campylotropis polyantha* at the two sites. Figure S3: Fold change (log_2_) of metabolites in leaves and roots of *Campylotropis polyantha* between the two sites and between leaves and roots.
