# Supplementary material for: A Preliminary Study on the Whole-Plant Regulations of the Shrub Campylotropis polyantha in Response to Hostile Dryland Conditions
Source: Metabolites. 2024 Sep 13;14(9):495. doi: 10.3390/metabo14090495 (PMC11433755; doi:10.3390/metabo14090495)
Supplement: Supplementary file 1 [file metabolites-14-00495-s001.zip › metabolites-3175695-supplementary.pdf]

Supplementary materials

Table S1 List of identified compounds in leaves and roots of *Campylotropis polyantha*.

Compounds	Retention time (min)	Groups
NA114002	12.09	Unknown compounds
Compound_13	13.03	Unknown compounds
Compound_14	14.12	Unknown compounds
Benzoic acid	14.83	Aromatics
Un-cgl-028	15.38	Unknown compounds
Compound_28	15.55	Unknown compounds
Phosphoric acid	15.69	Phosphrous compounds
Proline	16.12	Amino acids
Lumichrome	17.00	N-compounds
Pyrrole-2-carboxylic acid	17.49	N-compounds
Serine	17.75	Amino acids
Ethanolamine	18.39	N-compounds
Histidinol	18.54	N-compounds
Glyceric acid-2-phosphate	18.71	Phosphrous compounds
N-cinnamoyl glycine	19.84	Aromatics
Malic acid	20.56	Organic acids
Un-cgl-029	21.14	Unknown compounds
4-Aminobutanoic acid	21.26	Amino acids
2-oxo-Gulonic acid	21.52	Organic acids
Erythronic acid	21.81	Organic acids
Xylulose	22.66	Monosaccharides
Glutamic acid	23.13	Amino acids
Un-cgl-040	23.36	Unknown compounds
cis-4-hydroxy-Proline	23.63	N-compounds
Asparagine	24.16	Amino acids
Levoglucosan	24.83	Monosaccharides
Putrescine	25.20	N-compounds
Ribonic acid	25.83	Organic acids
Arabinonic acid	26.08	Organic acids
NA192001	26.31	Unknown compounds
1-Eicosanol	26.64	Fatty alcohols
Ornithine	26.74	Amino acids
3,4-Dihydroxybenzoic acid	26.76	Aromatics
Arginine	26.89	Amino acids
Citric acid	26.95	Organic acids
D-Pinitol	27.36	Polyols
Dehydroascorbic acid	27.45	Organic acids
Quinic acid	27.71	Organic acids
Fructose	28.01	Monosaccharides
Sorbose	28.18	Monosaccharides
Glucose	28.45	Monosaccharides
Mannose	28.70	Monosaccharides
Galactose	28.73	Monosaccharides
Mannitol	28.90	Polyols
Compound_99	29.01	Unknown compounds
Gallic acid	29.12	Aromatics
Ononitol	29.24	Polyols
Gulonic acid	30.00	Organic acids
Allantoin	31.21	N-compounds
myo-Inositol	31.37	Polyols
Sorbitol	31.78	Polyols
Idose	31.84	Monosaccharides

D226501	32.41	Unknown compounds
D227284	32.89	Unknown compounds
Panthenol	33.42	N-compounds
Glycerol	34.28	Polyols
Glycerolaldopyranosid	34.74	Sugar derivates
Benzoyl-beta-D-glucoside	34.75	Aromatics
Maltotriose	36.43	Trisaccharides
D256464	37.21	Unknown compounds
2,4-Dihydroxy-butanoic acid	37.34	Fatty acid
beta-Alanine	37.73	Amino acids
Isopropyl-beta-D-thiogalactopyranoside	37.82	Sugar derivates
Maltitol	38.03	Polyols
Arbutin	38.23	Aromatics
NA259001	38.43	Unknown compounds
Un-cgl-122	38.79	Unknown compounds
Methyl-beta-D-galactoside	38.91	Monosaccharides
Sucrose	39.22	Disaccharides
alpha-D-Glucopyranosyl-(1,6)-D-mannitol	39.48	Sugar derivates
4-Hydroxy-2-methylquinoline	39.67	Aromatics
Trehalose	40.35	Disaccharides
Un-cgl-122	40.98	Unknown compounds
D-Cellobiose	41.12	Disaccharides
Threitol	41.34	Polyols
D204282	41.51	Unknown compounds
Catechin	41.72	Aromatics
Epigallocatechin	42.19	Aromatics
D285215	42.24	Unknown compounds
Guaiacylglycerol	42.63	Aromatics
Octadecan-1-ol	42.70	Fatty alcohols
Galactinol	43.46	Polyols
Palatinose	43.70	Disaccharides
Uridine	44.10	N-compounds
Homovanillic acid	44.52	Aromatics
Myricetin	44.90	Aromatics
Compound_225	45.00	Unknown compounds
Methyl-alpha-D-galactoside	45.21	Monosaccharides
Taxifolin	45.40	Aromatics
5-hydroxy-Tryptophan	45.76	Aromatics
D-Xylobiose	46.10	Disaccharides
NA278005	46.61	Unknown compounds
Raffinose	47.49	Trisaccharides
Ampelopsin	48.38	Aromatics
6-Kestose	53.16	Trisaccharides
Epicatechin	55.45	Aromatics
Trans-Aconitic acid	55.95	organic acid
Taxifolin	56.51	Aromatics

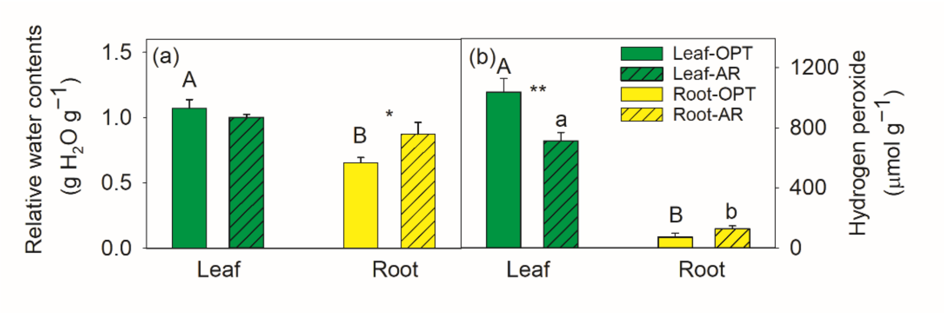

Figure S1. Water (a) and hydrogen peroxide (b) contents in leaf (green bars) and root (yellow bar) of *Campylotropis polyantha* at optimal site Cuojishan (OPT, without hatching) and arid site Lianghekou (AR, hatched bars). Asterisks indicate significant differences between the two sites within the same tissue (*, $p < 0.05$; **, $p < 0.01$). Different upper-case and lower-case letters indicate significant differences between leaf and root at OPT and AR, respectively. Data shown means ± SE (n = 6) on a dry weight basis.

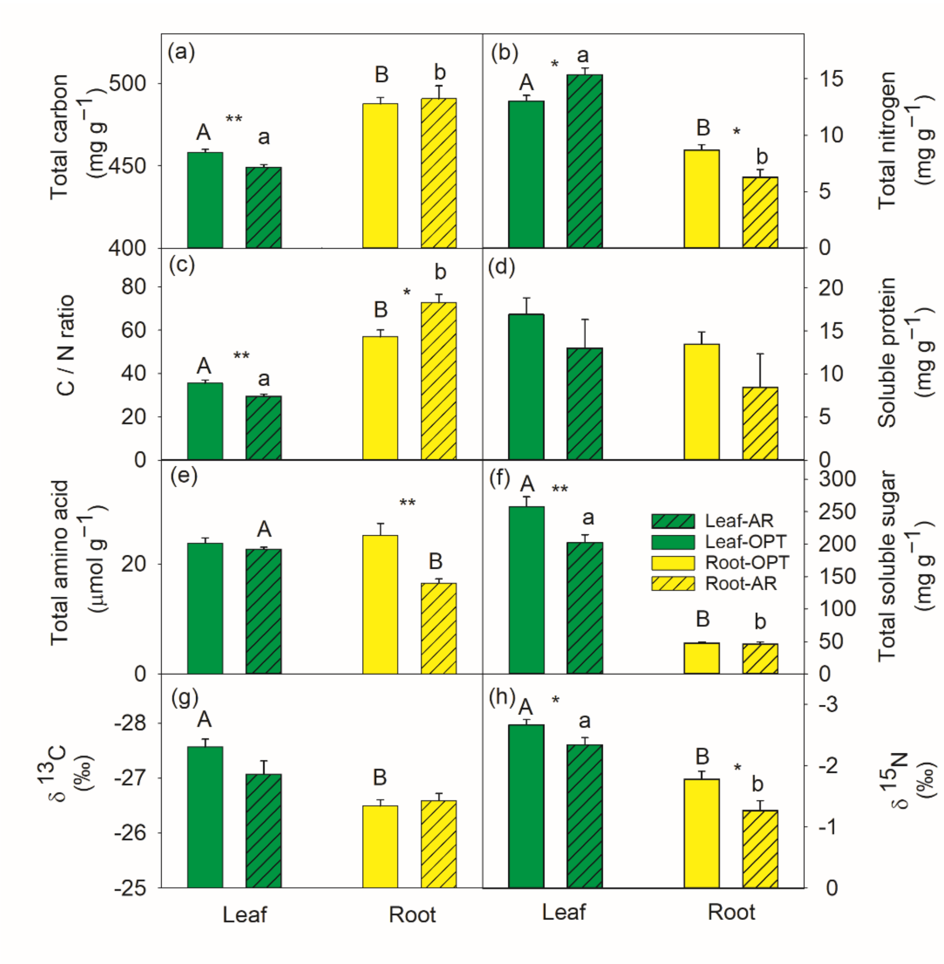

Figure S2. Total carbon (a), nitrogen (b) contents and their ratios (c), soluble protein (d), total amino acid (e), and soluble sugar contents (f), $\delta^{13}C$ (g) and $\delta^{15}N$ (h) in leaf (green bars) and root (yellow bar) of *Campylotropis polyantha* at optimal site Cuojishan (OPT, without hatching) and arid site Lianghekou (AR, hatched bars). Asterisks indicate significant differences between the two sites within the same tissue (*, $p < 0.05$; **, $p < 0.01$). Different upper-case and lower-case letters indicate significant differences between leaf and root at OPT and AR, respectively. Data shown means ± SE (n = 6) on a dry weight basis.

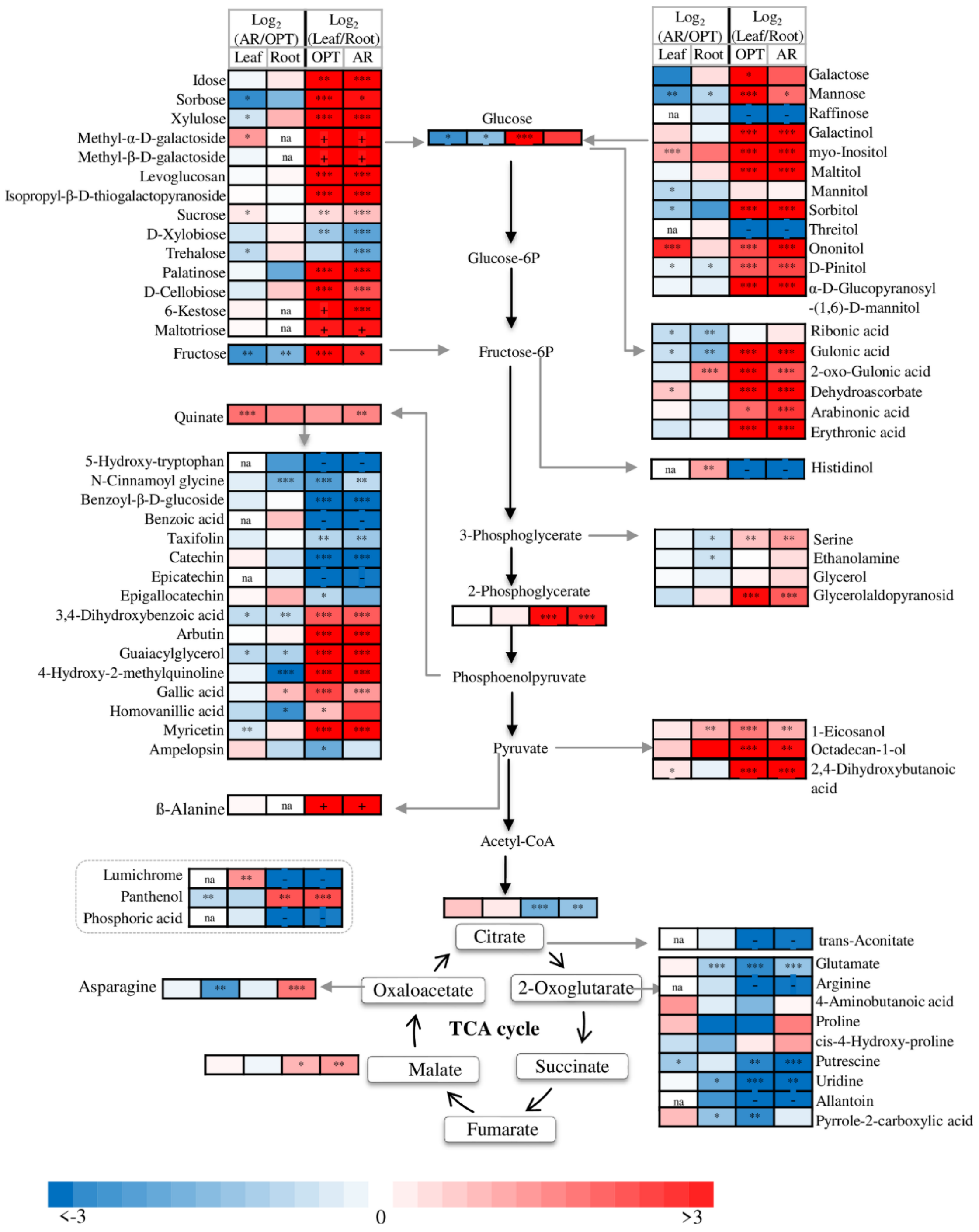

Figure S3. Fold change (log2) of metabolites in leaves (left panels) and roots (right panels) of *Campylotropis polyantha* between arid stie Lianghekou (AR) and optimal site Cuojishan (OPT), and between leaves and roots at OPT and AR, respectively. Asterisks indicate significant differences between sites within the same tissue, and between tissues within the same site (*, $p < 0.05$; **, $p < 0.01$; ***, $p < 0.001$; na, the metabolite was not detectable; +, metabolite only abundant in leaves; -, metabolite only abundant in roots). Data shown means ± SE (n = 6) on a dry weight basis.
